# Factors that dynamically affect provincial incidences of catastrophic health expenditure among middle-aged and elderly Chinese population-transition of disease financial risk protection from global to local

**DOI:** 10.1186/s12877-022-03432-6

**Published:** 2022-09-16

**Authors:** Xiyu Zhang, Wenqing Miao, Bing Wu, Yongqiang Lai, Mingli Jiao, Qi Xia, Chenxi Zhang, Wanxin Tian, Zhe Song, Linghan Shan, Lingqin Hu, Xinhao Han, Hui Yin, Xiaonan Cheng, Ye Li, Baoguo Shi, Qunhong Wu

**Affiliations:** 1 Research Center of Health Policy and Hospital Management, School of Health Management, Harbin Medical University, Harbin, 150086 Heilongjiang China; 2 School of Public Health, Harbin Medical University, Harbin, 150086 Heilongjiang China; 3 The second affiliated hospital of Harbin Medical University, Harbin, Heilongjiang China; 4 Department of Social Medicine, School of Health Management, Harbin Medical University, Harbin, Heilongjiang China; 5 The forth affiliated hospital of Heilongjiang University of Chinese Medicine, Harbin, Heilongjiang China; 6 Department of Biostatistics, School of Public Health, Harbin Medical University, Harbin, Heilongjiang China; 7 Department of Health Education, School of Health Management, Harbin Medical University, Harbin, Heilongjiang China; 8 Department of Economics, School of Economics, Minzu University of China, Beijing, 100081 China

**Keywords:** Spatiotemporal non-stationarity, Catastrophic health expenditure, Financial risk of disease, Regional policy, Health insurance

## Abstract

**Background:**

The high incidence of catastrophic health expenditure (ICHE) among middle-aged and elderly population is a major deterrent for reducing the financial risk of disease. Current research is predominantly based on the assumption of spatial homogeneity of nationwide population characteristics, ignoring the differences in regional characteristics. Thus, our study aimed to explore the impact of various influencing factors on the ICHE from a spatiotemporal perspective.

**Methods:**

We used data from the China Health and Retirement Longitudinal Study (waves 1 to 4), to conduct a retrospective cohort study across 28 provinces, from 2011 to 2018. We measured regional incidences of catastrophic health expenditure using methods recommended by the World Health Organization. Ordinary least squares (OLS) and geographical and temporal weighted regression (GTWR) were used as the global and local estimation models, respectively. The Fortheringham method was used to test the spatiotemporal non-stationarity.

**Results:**

National ICHE showed a gradual increase from 2011 to 2015, but suddenly decreased from 2015 to 2018, also showing the spatial heterogeneity. And the southwest area and Hebei showed persistently high ICHE (Qinghai even reached the highest value of 27.5% in 2015). Out-of-pocket payment, gross domestic product, PM_2.5_, ageing, incidence of non-communicable diseases and disabilities, number of nurses, and health insurance coverage in the global estimation passed the significance test, and the GTWR model showed a better model fit (0.769) than the OLS model (0.388). Furthermore, except for health insurance coverage, all seven variables had spatiotemporal non-stationarity among their impacts on ICHE.

**Conclusion:**

In this longitudinal study, we found spatiotemporal non-stationarity among the variable relationships, supporting regional governments’ adoption of regional-target policies. First, after the completion of universal health insurance coverage, the spatiotemporal non-stationarity of the prevalence of non-communicable diseases and disability and ageing should be the focus of the next phase of health insurance design, where improvements to compensation coverage and benefit packages are possible policy instruments. Second, the governance and causes of catastrophic health expenditure need to be laid out from a macro perspective rather than only from the individual/household perspective, especially for the potential impact of economic development, air pollution and nursing resources.

**Supplementary Information:**

The online version contains supplementary material available at 10.1186/s12877-022-03432-6.

## Background

Universal health coverage hopes that everyone has access to high-quality health services without being pushed to impoverishment, owing to the cost of paying for those services. Catastrophic health expenditure (CHE) is an official measurement indicator of financial risk. However, the World Health Organization (WHO) estimated that more than 150 million individuals were facing CHE in 2005 [1]. This number rapidly increased to 808 million in 2015 [2]. Furthermore, half of the global population does not have access to the healthcare they need [3, 4]. There is no doubt that a high CHE has always been a considerable deterrent in reducing the financial risk of disease.

China, the largest developing country in the world, has a severe financial risk of contracting the disease. The proportion of people aged 60 or over has increased by 5.44% in the last decade and reaching 13.5% in 2020 [5]. According to UN estimates, the number of Chinese elder population may further increase to 365,636,000 (26.1%) by 2050 [6], indicating an increasing disease burden of non-communicable diseases [7] (NCDs) and disabilities [8]. However, current social health insurance mechanisms have been insufficient to reduce the incidence of CHE (ICHE) among the middle-aged and elderly Chinese population [9, 10]. Further, it has become the short board for achieving UHC comprehensively, the determinants of which need to be further clarified.

There are several cohort studies on CHE determinants in middle-aged and elderly Chinese population [9, 11, 12]. The determinants of these studies include poverty, age, disability, NCDs, household size, inpatient or outpatient events, and social health insurance, considering time variation solely at the household level. All these conclusions drawn are based on the assumption that the characteristics of the national population are homogeneous. However, we need to pay special attention to China’s diverse geographical, socioeconomic, and ageing (AG) characteristics. The ICHE varies across regions nationwide as if it varies across countries worldwide [2, 13], with spatial non-stationarity. Unfortunately, despite several studies on the temporal non-stationarity of ICHE [9, 14, 15], the field of spatial non-stationarity is still lacking in all the existing studies related to ICHE. Additionally, whether the differences in factors’ influencing intensity exists between different provinces and whether the actual effect is diluted by the widely used global estimation methods remains to be seen. Therefore, we must further consider several issues. What factors dynamically affect the provincial ICHE among the middle-aged and elderly Chinese population? Is the relationship between these factors and ICHE the same throughout the country and in all provinces?

Our study makes the following contributions. First, we are the first to verify whether the occurrence of CHE is spatiotemporal non-stationary. Second, we constructed a more comprehensive framework for determinant analysis of ICHE by combining spatial and temporal perspectives. The impact of environmental dimension on ICHE was confirmed for the first time. Third, this study used geographical and temporal weighted regression (GTWR) as its primary method to further reduce the bias caused by ignoring the spatiotemporal position of the study sample. It considered strong relevance based on close spatiotemporal distance, while depicting the temporal and spatial evolution characteristics of the influencing factors of CHE for middle-aged and elderly population in China in eight years. Fourth, as the samples evolved from points to areas, the research units changed from households to regions, indicating that these local results are more instructive for regional policymaking without being diluted by global estimation.

To gain a better understanding of local spatiotemporal variation of ICHE and further formulate targeted policies to update health care systems in China, we conducted this retrospective cohort study from 2011 to 2018 by emphasizing the association among ICHE, out-of-pocket payment (OOP), socioeconomic factors, health service access, and health insurance coverage, under the consideration of spatiotemporal non-stationarity.

The remainder of this paper is organized as follows. First, according to the WHO estimation method, we calculated the CHE of every household and then converted the CHE and covariates into regional units. Second, we used global ordinary least squares (OLS) and GTWR with spatiotemporal location. We compared the results of the two models and tested for spatiotemporal non-stationarity. Third, we targeted the change characteristics of the temporal and spatial influencing factors affecting ICHE in households with middle-aged and elderly individuals. Finally, according to the above results, an evidence-based basis for improvement strategies is provided.

## Methods

### Sample selection

The China Health and Retirement Longitudinal Study (CHARLS) was conducted by the National Development Research Institute of Peking University. It is a longitudinal survey and is considered to be the most representative nationwide survey of the middle-aged and elderly population in mainland China. CHARLS conducted its national baseline survey from 2011 to 2012, Wave 2 was conducted in 2013, Wave 3 was conducted in 2015, and Wave 4 was conducted in 2018. The baseline survey included two person per household (aged 45 years or older), and in total involved 17,708 individuals living in 10,527 households across 28 provinces in China. However, we only removed households without subsistence food expenses, owing to their prime importance to CHE estimation and differences from common sense. We retained 16,161 individuals in 9224 households from the baseline survey, 15,320 individuals in 8662 households from Wave 2,17,763 individuals in 9977 households from Wave 3, and 17,408 individuals in 10,080 households from Wave 4. This cohort study included 28 provinces from 2011 to 2018 (Additional file 1: Appendix Fig. 1).

### Assessment of CHE

We employed CHE as a proxy to describe the financial risk of utilizing health services. CHE was accessed based on the definition of WHO [16], that is, the situation when OOP of a household on health equals or exceeds 40% of its capacity to pay. Household size, OOP, living budget, income, and food expenditure (*food*_*h*_) were extracted from the CHARLS questionnaire. The maximum value between the living budget and income (*exp*_*h*_) was calculated to better assess the consumption ability. *food*45 and *food*55 were defined as the food expenditure shares of *exp*_*h*_ at the 45th and 55th, respectively.

We calculated the food expenditure share by dividing the household’s food expenses by *exp*_*h*_, that is, *foodexp*_*h*_. The household equivalence scale was used rather than the actual household size due to household consumption size, which is given below:

1 $${eqsize}_h={hhsize}_h^{\beta }$$

where *hhsize*_*h*_ is the household size from the CHARLS questionnaire, and parameter *β* equals 0.56, according to 59 countries’ household survey data [13].

Equivalized food expenditure (*eqfood*_*h*_) was calculated by dividing household food expenditure by *eqsize*_*h*_. The poverty line (*pl*) and subsistence expenditure (*se*_*h*_) were then generated based on the *eqfood*_*h*_:

2 $${eqfood}_h=\frac{food_h}{eqsize_h}$$

3 $$pl=\frac{\sum {w}_h\ast {eqfood}_h}{\sum {w}_h}, where\ food45<{foodexp}_h< food55$$

4 $${se}_h= pl\ast {eqsize}_h$$

A household’s capacity to pay is the non-subsistence spending of a household. Depending on whether self-production, coupons, food subsidies, and other non-cash means of food consumption are considered, two estimation methods are given below:

5 $${\displaystyle \begin{array}{c}{ctp}_h={\mathit{\exp}}_h-{se}_h\mathrm{if}\ {se}_h<={food}_h\\ {}{ctp}_h={\mathit{\exp}}_h-{food}_h\ if\ {se}_h>={food}_h\end{array}}$$

Then, the burden of health OOP (*oopctp*_*h*_) was calculated as the health OOP share of *ctp*_*h*_.

6 $${ oop ctp}_h=\frac{oop_h}{ctp_h}$$

Finally, we considered a household incurring CHE if *oopctp*_*h*_ equals or exceeds 0.4, according to the WHO definition as follows:

7 $${\displaystyle \begin{array}{c}{cata}_h=1\ \mathrm{if}\ {oopctp}_h>=0.4\\ {}{cata}_h=0\ \mathrm{if}\ {oopctp}_h<0.4\end{array}}$$

### Statistical analysis

From the perspective of previous multi-country analyses [2, 13], the main determinants of CHE can be represented by OOP, economic level, health service demand and use, and failure of social mechanisms to pool financial risks. We used regional gross domestic product (GDP) to reflect the economic development, particulate matter (PM_2.5_) to reflect the air pollution, and used the proportion of population aged 65 or over, incidence of NCDs, incidence of disability, and number of nurses per thousand persons to reflect the health service demand and provision. Health insurance coverage was adopted to reflect the success or failure of social mechanisms in pooling financial risks. In addition, regional development strategies undertaken by the central government may have impacts on ICHE via economic development, health service provision, and health insurance coverage and therefore need to be added as a control variable [17]. Some studies [18] reported the association between geographical location and regional development strategies [19, 20]. We therefore employed the geographical subdivision as the proxy variable to replace this unobservable variable, further dealing with endogeneity caused by omitted regional development strategies and unobserved heterogeneity caused by population migration. Finally, we introduced a seven-category variable based on geographical area as a control variable to distinguish between regional development strategies [17, 21]. All individual and household indicators were converted into regional indicators for further spatiotemporal analysis (Additional file 1: Appendix Fig. 1). Descriptions of all the indicators are shown in Table 1 and Additional file 1: Appendix Table 1. In future work, we will develop global OLS and GTWR models to measure the potential effects caused by spatiotemporal non-stationarity.

**Table 1:** Description of Independent Variables

Dimension	Variable	Data Source	Unit
**OOP**	X_1_: Average Out-of-pocket Payment (OOP)	CHARLS	Yuan
**Socioeconomic factors**	X_2_: Gross Domestic Product (GDP)	China Statistical Yearbook in 2012, 2014, 2016 and 2019	100 Billion Yuan
**Air pollution**	X_3_: Annual average PM_2.5_ concentration in the one year before the survey (PM_2.5_)	Global Burden of Disease Collaborative Network, Global Burden of Disease Study 2019	ppb
**Health Service Demand**	X_4_: Proportion of Population Aged 65 or Over (AG)	CHARLS	%
	X_5_: Prevalence of Non-communicable Diseases (NCDs)	CHARLS	%
	X_6_: Prevalence of Disability (Disability)	CHARLS	%
**Health Service Provision**	X_7_: Number of Nurses Per Thousand Persons (Nurses)	China Health and Family Planning Statistical Yearbook in 2012 and 2016 Statistical Yearbook of China Tertiary Industry in 2014 and 2019	N/A
**Health Policy**	X_8_: Health Insurance Coverage (Insurance)	CHARLS	%
**Control Variable**	Group: Group according to the geographical subdivision		

*CHARLS* China Health and Retirement Longitudinal Study

#### Global OLS model

First, the global OLS model was used for the analysis without any spatial and temporal considerations. The equation is as follows:

8 $${Y}_i={\beta}_0+\sum_k{\beta}_k{X}_{ik}+{\varepsilon}_ii=1,\dots, n$$

#### GTWR model

GTWR is a spatiotemporal statistical method and generally fits local regression models in space-time based on distance-decay effects, i.e., data of their own and surrounding units are considered. GTWR further considers timestamps and spatial locations based on the OLS regression model, allowing for specific parameter estimation for each spatiotemporal unit. It considers both temporal and spatial heteroskedasticity and outperforms OLS in the model accuracy for the sample data. Further, it has been used widely in house pricing and air pollution studies [22–25].

Based on previous ICHE studies, we found that ICHE is a disease financial risk indicator that varies by spatiotemporal location. With the increasing accuracy requirements of ICHE studies, its influencing factors should be analyzed with full consideration of spatiotemporal variation. Making full use of spatial panel data, GTWR helps provide direct evidence for regional policymaking. Especially in the case of health policy, regional guidelines need to take full account of their characteristics and those of neighboring regions. GTWR is expected to be one of the effective tools for regional policymaking.

Similar to OLS, GTWR takes timestamps into the origin coordinate framework and constructs a weight matrix to account for spatiotemporal non-stationarity in the parameters. The timestamp was addressed by setting it to a certain year. Therefore, the GTWR model and its parameter estimation can be expressed as:

9 $${Y}_i={\beta}_0\left({u}_i,{v}_i,{t}_i\right)+{\sum}_k{\beta}_k\left({u}_i,{v}_i,{t}_i\right){X}_{ik}+{\varepsilon}_i\ i=1,\dots, n$$

10 $$\hat{\beta}\left({u}_i,{v}_i,{t}_i\right)={\left[{X}^TW\left({u}_i,{v}_i,{t}_i\right)X\right]}^{-1}{X}^TW\left({u}_i,{v}_i,{t}_i\right)Y$$

where *W*(*u*_*i*_, *v*_*i*_, *t*_*i*_) denotes a new matrix whose diagonal elements are the spatiotemporal distance functions of (*u*_*i*_, *v*_*i*_, *t*_*i*_) and weights when calibrating a weighted regression adjacent to spatiotemporal unit *i*. An adaptive kernel and AICc [26] were used in our GTWR model.

#### Test for spatiotemporal non-stationarity

Many non-stationarity assessment techniques have been further employed to test its significance. However, Fortheringham [23, 27], as the frontier in applying the geographical weighted regression model, provided a technique to twice compare the standard errors of OLS estimates with the interquartile range of GTWR to assess the non-stationarity of coefficient estimated. Larger values of the latter are considered to indicate significant spatial non-stationarity.

## Result

### Range of ICHE variation from 2011 to 2018

**Fig. 1:**
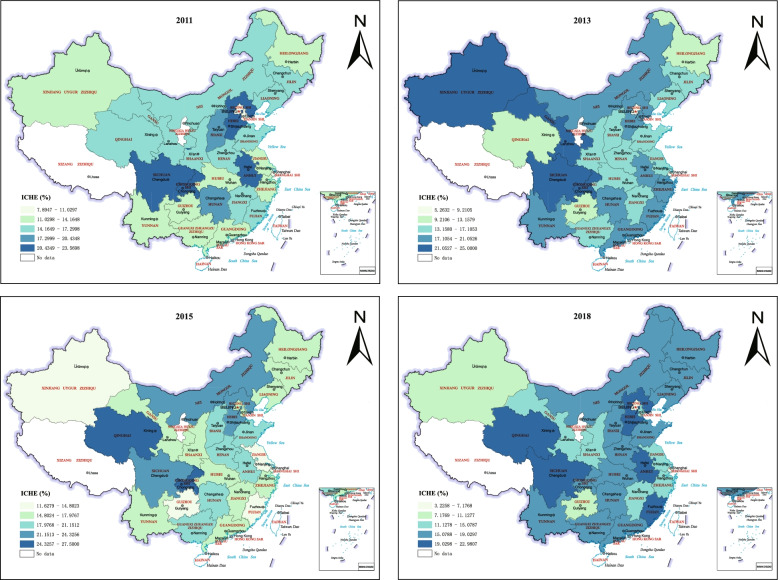
Spatiotemporal distribution for incidence of catastrophic health expenditure from 2011 to 2018 among middle-aged and elderly Chinese population

We calculated the ICHE nationwide across 28 provinces in mainland China using Ke Xu’s method. Figure 1 shows the distribution of provincial ICHE and its temporal variations. According to the results, temporal variations in ICHE were found. The ICHE shows a gradual increase from 2011 to 2015 but suddenly decreased from 2015 to 2018. Individually, the ICHE of Beijing has decreased from 9.804 to 3.226% from 2011 to 2018, with the most drastic decline, whereas Qinghai reached its highest value of ICHE (27.5%) in 2015. In addition, the southwestern area (including Qinghai, Sichuan, and Chongqing) and Hebei in China shows persistently high ICHE. The ICHE in 2015 shows great differences among provinces, whereas in other years, the difference is relatively small.

### Results of global estimation and the multi-collinearity test

OLS was first used for global estimation, and multi-collinearity tests of all independent variables were then performed. The results are presented in Table 2. The F-test result indicates this model is significant at 99% level, while the global estimation can solely explain 38.8% of the variation in ICHE based on R^2^.

**Table 2:** Global OLS regression result

ICHE	Coefficients (95% CI)	St.Err.	*p*-value	VIF
X_1_	.007[.003, .011]	.002	0	1.50
X_2_	.004 [−.001, .009]	.002	.103	1.44
X_3_	.028[−.013, .253]	.016	.071	1.20
X_4_	.120[−.066, .072]	.067	.077	2.49
X_5_	.003[−.067, .072]	.035	.935	1.69
X_6_	.194 [.026, .362]	.085	.024	1.64
X_7_	−.319[−.446, −.192]	.064	0	2.75
X_8_	−.051[−.137, .034]	.043	.236	1.17
Control_group	−.094[−.270, .458]	.183	.610	1.32
Constant	25.148 [7.229,27.262]	5.050	.001	
F-test	7.18			
Prob>F	0.000			
R^2^	0.388			
AICc	611.837			

*ICHE* Incidence of catastrophic health expenditure, *OLS* Ordinary least squares, *St.Err.* Standard error, *AICc* Corrected Akaike information criterion

Furthermore, OOP and nurses are considered significant at 99% level, disability is deemed significant at 95% level, and PM_2.5_ and AG are considered marginally significant at 90% level. In addition, all the independent variables passes the multi-collinearity test and are considered for local estimation.

### Results of the GTWR model and the spatiotemporal non-stationarity test

The GTWR model was tested further (Table 3). It should be noted that the residual sum of squares decreases from 1293.203 to 492.273, and R^2^ increases from 0.388 to 0.769 largely between OLS and GTWR, although the AICc increases slightly. This demonstrates that the local estimation of GTWR is better than the global estimation of OLS for this spatial cohort study, indicating that spatiotemporal non-stationarity helps to explain the variety of data. Introducing geographical subdivisions as control variable also makes the further improvement of the goodness of fit of the model (Additional file 1: Appendix Table 2).

**Table 3:** Comparison of Global OLS and GTWR models

	Global OLS	GTWR
**Bandwidth**		42
**Residual Sum of Square**	1293.203	492.273
**AICc**	611.837	622.243
**R**^**2**^	.388	.769
**Adjusted R**^**2**^		.749

*GTWR* Geographical and temporal weighted regression, *OLS* Ordinary least squares

**Fig. 2:**
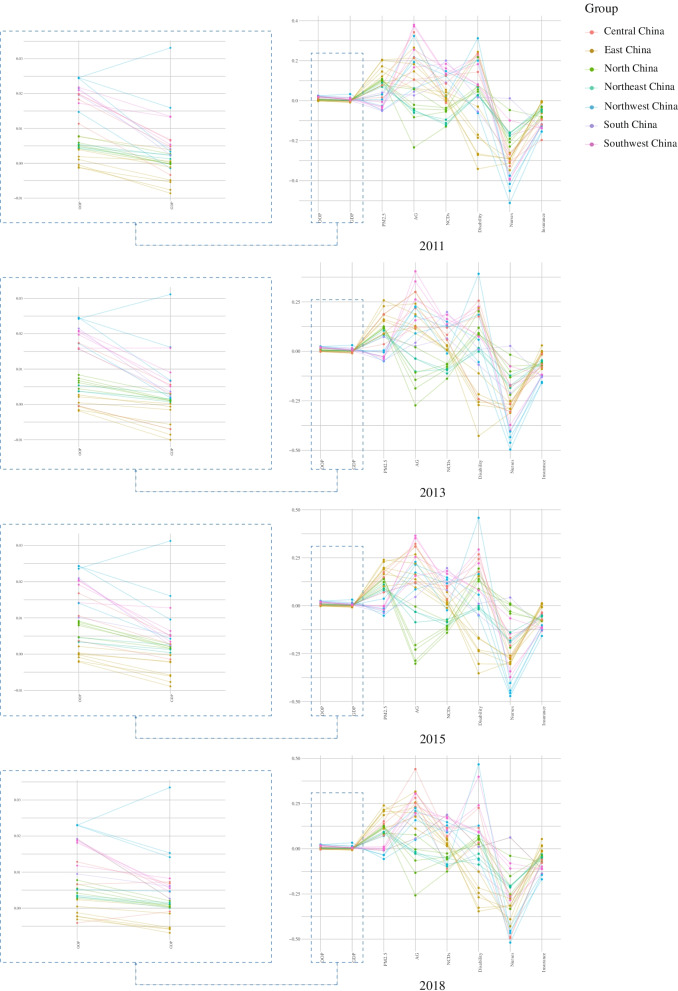
Variation trend of coefficients of all variables among spatiotemporal units

Furthermore, this study applied GTWR as the final estimation model. The coefficient variation distribution of the eight independent variables (the control variable is not listed) is shown in a parallel coordinate plot [28] (Fig. 2). It is worth noting that the number of nurses per thousand persons and health insurance coverage have almost always been protective factors against ICHE.

Then spatiotemporal non-stationarity tests were adopted to test whether differences exist in eight independent variables among spatiotemporal units (Table 4). The coefficients of OOP, GDP, PM_2.5_, AG, NCDs, disability, and nurses exhibited extra local variation beyond purely sampling expectations. Therefore, health insurance coverage was considered a global variable without spatiotemporal non-stationarity.

**Table 4:** Spatiotemporal non-stationarity tests of independent variables

Variable	Interquartile Range	2 × SE (OLS)	Extra local variation
X_1_	0.004	0.016	Y
X_2_	0.004	0.006	Y
X_3_	0.032	0.140	Y
X_4_	0.134	0.233	Y
X_5_	0.070	0.173	Y
X_6_	0.170	0.223	Y
X_7_	0.128	0.164	Y
X_8_	0.086	0.083	N

*SE* Standard error, *OLS* Ordinary least squares

### Description of the variety spatiotemporal trends

As shown in Fig. 2, the distribution of the coefficients of the seven independent variables with spatiotemporal non-stationarity shows a significant difference between regions.

#### OOP

It is estimated that the positive impact of OOP decreases gradually from west to east spatially and temporally from 2011 to 2018. However, some spatiotemporal units differs. For instance, the impact of OOP on the ICHE in North China increases from 2011 to 2015 and decreases after 2015.

#### GDP and PM_2.5_

For GDP, which describes economic development, fascinating conclusions can be drawn. In relatively economically developed East China, GDP is a protective factor for ICHE, while it is a weakening risk factor in some regions, such as Central China and Northeast China. The impact of GDP on ICHE may demonstrate the complexity of the health effects of economic development.

Air pollution is a topic of interest. Spatially, its positive impact on the ICHE decreases from east to west. Moreover, its positive impact on ICHE gradually increases, but decreases in parts of East China and North China after 2015.

#### AG, NCDs and disability

For AG, most of its positive impact is concentrated in regions other than North China and Northeast China. Temporally, the positive impact of AG increases from 2011 to 2018 in East China, Central China, and South China but decreases after 2013 in Northwest China and Southwest China.

Most of the positive impacts of NCDs on ICHE are concentrated in regions similar to AG. Temporally, the positive impact of AG increases from 2011 to 2018 in East China but decreases after 2013 in Central China, South China, Northwest China, and Southwest China.

However, the spatial distribution of the positive impacts of disability differs from that of the first two and exists in regions other than East and Northeast China. However, it is noteworthy that it was significantly enhanced in Southwest China and Shaanxi Province. Temporally, most regions show a weakened positive impact of disability on ICHE after 2015, but this does not apply to Southwest China and Shaanxi Province. In these two regions, the positive impact of disability on ICHE maintains a possible upward trend.

#### Nurses

For nurses, we found a clear downward trend in all regions nationwide except Northeast China, especially until 2018. Furthermore, the widespread strong negative impact in Northwest China should not be ignored.

## Discussion

In this study, the spatiotemporal relationship between ICHE and influencing factors was locally estimated for each province in China using the GTWR model. As far as we are concerned, this study is the first application of the GTWR model in health policy and made up the major blank area of spatial non-stationarity in the existing time-series studies. Based on the GTWR model, the results in this study confirmed the existence of spatiotemporal non-stationarity of OOP, economic factors, health service demand, provision and provided evidence for the formulation of related region-targeted policies.

### Existence of the spatiotemporal heterogeneity of ICHE among the middle-aged and elderly Chinese population in recent years

The ICHE among the middle-aged and elderly Chinese population is spatiotemporally heterogeneous. Temporally, the ICHE in this study increases from 2011 to 2015 and decreased from 2015 to 2018, showing similar trends in the previous literature [29]. In fact, with the frequent occurrence of NCDs and physical multimorbidity in the elderly in recent years [9], the economic burden of diseases among middle-aged and elderly residents in China has gradually increased. Under the background of such high demands and utilization of diseases, China’s ICHE could still show a downward trend in time series, with the implementation of a series of health reform measures in China, such as the establishment and deepening of multi-level health insurance systems and remarkable achievements in the essential drug system [30, 31]. The timeline of China’s series of health reform measures almost matches that of ICHE reduction, which proves that health policies make outstanding contributions to the control of ICHE in China [32]. Spatially, the distribution of ICHE in this study is also markedly heterogeneous, which echoes much of the literature [33]. Southwest China and Hebei Province exhibits persistently high ICHE. NCDs is a major cause of CHE in households with the middle-aged and elderly population and its causes in China vary greatly between regions [34]. The burden of NCDs is high in several provinces in the Southwest China, where poor lifestyle habits due to ethnic customs, urbanization, and westernization [35, 36] have raised the risk of developing NCDs. As for Hebei Province, it is facing serious air pollution problems which may further cause NCDs. In addition, the relatively low economic level [37] and severe AG problems [38] may further worsen the financial risk. Therefore, the high regional ICHE shows the diversified causes and needs to be addressed in a targeted manner depending on the causes.

### Health insurance system: after the achievement of population universal coverage, special attention to vulnerable groups and targeted policies are still the key point

Health insurance is currently the most effective policy tool for controlling ICHE. China has made remarkable achievements in approaching the goal of near-universal health insurance coverage, although its depth is in need of enhancement [39]. For depth, we focused on two main questions: “how much protection to provide” and “who to protect.”

First, the spatiotemporal non-stationarity of the impact of OOP on ICHE can be seen in this study and previous literature [40], which indicates the answer to “how much protection to provide” is not the only. Our results further show that the positive impact of OOP diminishes from west to east, similar to the regional differences in China’s economic development. One possible explanation is that per capita income is higher in economically developed regions [41]. Thus, households with middle-aged and elderly population living in these regions are more resilient to the financial risk of disease. Furthermore, different geographical regions are accompanied by development differences, which leads to many differences in the design of health insurance systems, financing systems, benefit packages, and health insurance governance. A “one-size-fits-all approach” to frame regional health insurance policies would not be sufficient [42]. At the same time, the overall decreasing trend of OOP coefficients temporally affirms the role of health insurance under healthcare reform. However, parts of North China may need to reverse the upward trend in deepening reforms.

Second, the key to answering the question “who to protect” can be found in the results for AG, NCDs, and disability.

The spatiotemporal non-stationarity [42] of NCDs and AG have been identified. For NCDs and AG, most of the positive impacts are spatially concentrated in regions, except for North China and Northeast China. Furthermore, they all show an increasing trend in East China from 2011 to 2018 and a decrease in Southwest China after 2013. The burden of NCDs increases along with AG [43] and health insurance should therefore strengthen the financial protection for NCD patients and the elderly. Our results can be used to test past regional policy implementations, but it seems that some regions are doing far less well than we thought. Taking East China as an example, previous studies have also shown that some provinces in East China are risk factors for the health of the elderly [38]. High-risk factors for NCDs in East China may include the harmful use of alcohol and overweight or obesity [44], which confirms the impact of urbanization and economic development on NCDs. Some studies have found an increasing proportion of elderly patients hospitalized with NCDs in East China [45].

Although a consensus has been reached that NCDs have become the focus of health insurance, there are still differences in economic protection among regions, based on the development status of existing health insurance funds, health demand, and economic level. How can we further deepen the protective effect of the health insurance system on the elderly with multiple vulnerable characteristics? It is breaking the fragmentation of health insurance schemes, further broadening the benefit package of NCDs, and enriching compensation schemes that have become breakthroughs for all regions to reduce the ICHE. It is imperative and wise for all regions to first realize the coverage of inpatient services for elderly patients with NCDs [46], which has directly brought about obvious financial risk mitigation effects. However, it is not friendly enough for patients with NCDs who do not often require hospitalization [33]. Considering the regional disease characteristics of the middle-aged and elderly population and the nearly saturated financial protection system for inpatient services, local governments should to some extent further expand the economic protection system for outpatient services.

Few literatures have focused on the non-stationarity of the spatiotemporal relationship between disability and ICHE. Furthermore, much of the relevant literature is based on the spatiotemporal stationarity hypothesis [47]. Temporally, we found that most regions show a weakened positive impact of disability on ICHE after 2015, but in Southwest China and Shaanxi Province, the positive impact of disability on ICHE still maintains a possible upward trend. Additionally, it was significantly enhanced spatially in Southwest China and Shaanxi Province. Our study further demonstrates the variability in the regional characteristics of disability and economic protection policies. First, few policies specifically target people with disabilities [48]. Second, disability in the middle-aged and elderly Chinese population is often caused by the disease. However, current health insurance policies in most provinces only focus on the financial risk in the disease stage (including inpatient and outpatient services), yet the disability stage (including the rehabilitation service) is the most likely to be problematic. For example, Yunnan province, located in Southwest China, has the second highest number of the poor in China and accounts for 3.78% of national disabled population. The results of this study show the increasing impact of disability on ICHE in Yunnan Province, which can be attributed to insufficient government finances, incomplete coverage, inadequate compensation, and the lack of rehabilitation services, especially in rural areas. Current health insurance benefit packages are inadequate and are unable to financially protect the disabled at all stages. There are over 85 million people with disabilities in China, 90% of whom have rehabilitation needs. However, only slightly more than 10 million people have access to rehabilitative care. Furthermore, 130 million people with NCDs also have this rehabilitation requirement. On the one hand, 50% of doctors, 33% of therapists, and nearly 15% of nurses had a bachelor’s degree or higher; on the other hand, rehabilitation resources are unevenly distributed among regions and between urban and rural areas, limited to clinical settings, and not extended to family, community, and other social settings [49]. Therefore, the government should formulate health insurance targeting people with disabilities, focus on the financial protection of the rehabilitation demands of the disabled, and establish a good connection from the disease stage to the disability stage from the perspective of policymaking. Furthermore, both the expansion of social rehabilitation networks and provision of appropriate payment and pricing policy support are required.

One issue that needs to be clarified is that outliers contrary to common sense (especially in health service demand and utilization) may be due to the non-utilization of health services resulting from poverty, which is particularly common in regions with low levels of economic development [14, 50, 51].

### The achievement of the economic development on ICHE: high economic level and the dark side

The impact of economic factors on ICHE is bidirectional and spatiotemporally non-stationary, and often diluted by global estimation.

We used PM_2.5_ to measure the impact of air pollution due to economic development. We find that its positive impact diminishes from east to west, corresponding to the differences in economic development in China. Some studies [52] have also found a correlation between the two, suggesting that low PM_2.5_ concentrations tend to be concentrated in less economically developed regions. Thus, air pollution problems in economically developed regions exacerbate health demand, raising concerns regarding increased ICHE.

Economic development provides improved living standards, increases investment in health, and indirectly influences health through education; however, at the same time, economic development brings environmental pollution, poor lifestyles, psychological problems, and AG. In our study, GDP was a protective factor for ICHE in relatively economically developed East China and a weakening risk factor in Central China and Northeast China. GDP is considered to have a complex relationship with health.

Although possible urbanization exacerbates the risk of NCDs in East China, the improvement in the economic capacity of regional residents as a result of economic development is substantial, consistent with our earlier discussion. According to the current results, GDP growth has brought more disease and issues such as AG to relatively underdeveloped regions, thereby obscuring its benefits. However, we observed a decreasing positive impact in Central and Northeast China, illustrating the improvements achieved in these regions in the past. One possible reason for this is the enormous contribution China has made to eradicate poverty over a long period. The Chinese government has committed to poverty reduction over the past few decades, decreasing the number of the poor from 98,990,000 to 1660 from 2012 to 2018. It has further eliminated absolute poverty by 2021 [53].

Previous studies have suggested that health insurance policies that take into account not only health expenditure but also income should be developed [54]. Similar to them, our study also argues for the possible impact of economic development on the ICHE based on the regional level. Vietnam and Turkey provide very good precedents, and their success in achieving UHC can be attributed to the provision of health benefits to low-income households. In addition to focusing on household characteristics, whether CHE occur should also focus on macro characteristics. The pattern of regional health insurance, economic development and ecological environment may be the factors that really play a major role in ICHE controlling especially in the context of the spatiotemporal non-stationarity.

### The power of nursing: attention to both quantity and quality

As part of the health human resource, nurses are crucial for reducing ICHE in China. This study found that the impacts of nurses on ICHE are spatiotemporally non-stationary and that these impacts are almost all negative. Few studies have provided direct evidence for the association between nurses and ICHE, while many studies have proven the relationship between nurses and health [55]. Nurses play an important role in all stages of disease prevention, treatment, and rehabilitation. An increase in the number and quality of nurses could radically curb the massive deterioration of the disease in the region and avoid elevated ICHE. When the number of patients cared for by each nurse decreased by one, the mortality within 30 days, the readmission rate within seven days after discharge decreased by 7%, and the length of hospitalization decreased by 3% [56], indicating less medical expenditure. Improving the regional nurse-patient ratio and enhancing nurse education are options with a high cost-benefit ratio, according to the local health service demand. Nursing is even more important for middle-aged and elderly populations, which have a high prevalence of NCDs. Therefore, it is also essential to consider the number of middle-aged and elderly NCDs patients in the regional standard nurse-patient ratio, especially in Northeast China and Northwest China. In addition, offering high payment rates in under-served regions is also considered an effective measure according to WHO suggestion [57].

### Implication to policymakers and scholars

First, perspectives need to be updated from individuals/households to regions. With the process of socioeconomic development and poverty elimination, residents’ demand for health is not only to have access to obtain health services, but also to obtain them without economic burden. Previous studies on the identification of factors affecting CHE are almost all based on the individual and household level, further targeting the characteristics of vulnerable groups with priority intervention. However, interventions based on the perspective of individuals and households cannot cover the impact of economic development, social welfare system and environmental factors on health and economic burden of disease. It is not in line with the current and future demands for multidimensional and deep resistance to disease financial risks. The identification of vulnerability characteristics of regional CHE is an inevitable path for effective governance in the next stage. It is urgent to step away from the household level and conduct collaborative governance on the impact mechanism that drives the occurrence of CHE from a regional and national perspective. Moreover, the influencing factors of CHE usually have the characteristics of time inheritance and path dependence of institutional change. Therefore, capturing the time cycle of the characteristics of regional CHE is to ensure the accuracy of intervention policies. By upgrading the research perspective to spatiotemporal perspective, it provides accurate evidence for countries to formulate differentiated and targeted governance strategies.

Second, the theoretical framework needs the extension with the macro social structural factors. With the development of AG and high burden of NCDs, people must pay attention to the influencing factors causing CHE. Among these influencing factors, macro social structural factors may play an important role. As these macro factors have caused a huge impact on public health and economic status, leading to the rise of regional ICHE. People began to realize that in response to these factors, individuals played a limited role, while regional governments began to occupy the main position. This requires further spatiotemporal monitoring and intervention on the impact of these factors on ICHE at the regional level, helping people get rid of potential financial risks of disease. Therefore, it is necessary to extend the current theoretical framework to help it shift its attention to macro factors.

Third, the intervention subjects of ICHE need further enrichment. In addition, the sources of these influencing factors also indicate that ICHE is not just an issue of the health system, while more departments need to participate in. In the above process, culture, politics, environment and economy may cause CHE through health, income or insurance. Therefore, the source of CHE may be diversified, and its intervention requires multi-agent collaborative governance.

Forth, the proper methodologies need to be introduced to find supportive evidence. These issues show great heterogeneity at the regional level, suggesting that it is not enough to focus on the distribution differences of health outcomes in the past. What is more important is the heterogeneity of the formation process of health outcomes, i.e., spatial non-stationarity. Interestingly, it not only presents a shift in focus, but also puts forward higher requirements for the methodology. Therefore, we finally chose GTWR as the potential analysis method. Compared with most previous studies only making a visualization of ICHE, our study using GTWR shows variation among parameters in every spatiotemporal unit. The upgrading of methodology provides evidence for the above solutions based on social governance. Due to the varying-parameter assumption, the parameters estimated by GTWR show heterogeneity among different spatiotemporal units. These results are of great help to regional governments in identifying the priorities of governance.

In addition, in the real world, we tend to think that the social systems, culture and other factors in one region and surrounding regions are highly similar. However, these factors are often unobservable. After mixing different samples from multiple regions, they constitute the unobservability of the variation. This may not appear in GTWR in theory, as for each sample included in the local regression, they are closer to the center point (Geography believes that things close are more closely related). These factors avoid the unobservable part of the global regression to a certain extent due to the similarity of points included in the same local regression. Therefore, it is meaningful to use GTWR in a topic highly related to social factors such as health expenditure. For those potentially unobservable confounding factors, they constitute the spatiotemporal non-stationarity in these local regression models.

Our study has several limitations. First, current CHE calculation method ignores the groups that do not utilize health services and the ICHE in some groups is underestimated, especially in the poor. In the future, estimation of accurate regional ICHE based on macro data through applicable econometric methods (such as Newton-Cotes) could be considered. Second, this study lacks robust evidence for inferring causation because none of studies focused on the spatial non-stationarity of ICHE. However, at the same time, it also means that the spatiotemporal non-stationarity of ICHE proposed in this paper is innovative. Third, selective attrition may exist as in most CHARLS studies and population with serious health problems are more likely to drop-out. Another concern is that the CHARLS dataset is not sufficiently regionally representative. Data quality should be further enhanced, such as establishing some regionally representative databases and expanding the sample size. Forth, the use of self-reported NCDs and disability data is also a limitation of this study, which could be improved in the future via expanding data accessibility. Finally, the influencing factors involved in this study may not capture the comprehensive processes and the interaction effects towards ICHE disparities, therefore some omitted variables were inevitable. Methodology for dealing with non-liner models and models with more types of endogeneity need to be further developed.

## Conclusion

Based on the GTWR method, this study breaks the hypotheses of fixed coefficients and global stationarity. It explores the spatiotemporal non-stationarity of the relationship between various factors and ICHE, which can be used as an essential basis for regional policy reform.

First, this study called for extensive consideration of spatiotemporal non-stationarity in health. Based on the results of the model comparison in this study, we further expanded the interpretation of the global model by considering the coefficient of variation with spatiotemporal coordinates. Significant progress has been made after considering spatiotemporal non-stationarity, indicating that extensive related studies should consider it to further improve their quality.

Second, the existence of spatiotemporal non-stationarity in ICHE has far-reaching implications for subsequent related policy formulation.

Third, after the completion of universal health insurance coverage, the spatiotemporal non-stationarity of the prevalence of NCDs and disability and AG should be the focus in the future. Based on this, improvements to compensation coverage and benefit packages are possible policy instruments. For example, consider outpatient services for patients with NCDs, rehabilitation services for people with disabilities, and the financial risk of disease for the elderly in the subsequent design of health insurance. Considering the spatiotemporal non-stationarity of these factors together can help improve the propensity of health insurance policies.

Forth, economic development shows complex impacts on ICHE. Especially, air pollution, as one of its consequences, leads to higher ICHE. In contrast, the allocation of nursing resources helps to reduce the ICHE. This further suggests that the governance and causes of CHE should not be only at the individual/household level and need to be laid out from a macro perspective.

## Supplementary Information

**Additional file 1: Appendix Table 1.** Reference of indicators selection. **Appendix Table 2.** Comparison of GTWR model without geographical subdivisions and that with it. **Appendix Figure 1.** Flowchart of data process in this study.

## Data Availability

The data used in this study are proprietary data owned by Peking University. The data are not publicly available but are available to researchers from corresponding author by application to the Peking University for CHARLS Program (http://charls.pku.edu.cn/en).

## Abbreviations

CHE: Catastrophic health expenditure
WHO: World health organization
ICHE: Incidence of CHE
AG: Ageing
GTWR: Geographical and Temporal Weighted Regression
OLS: Ordinary least squares
CHARLS: China Health and Retirement Longitudinal Study
OOP: Out-of-pocket payment
GDP: Gross domestic product
PM_2.5_: Particulate matter
NCDs: Non-communicable diseases
