# Supplementary material for: Factors that dynamically affect provincial incidences of catastrophic health expenditure among middle-aged and elderly Chinese population-transition of disease financial risk protection from global to local
Source: BMC Geriatr. 2022 Sep 16;22:759. doi: 10.1186/s12877-022-03432-6 (PMC9479304; doi:10.1186/s12877-022-03432-6)
Supplement: Supplementary file 1 — Additional file 1: Appendix Table 1. Reference of indicators selection. Appendix Table 2. Comparison of GTWR model without geographical subdivisions and that with it. Appendix Figure 1. Flowchart of data process in this study. [file 12877_2022_3432_MOESM1_ESM.zip › Appendix.docx]

**Appendix Table 1. Reference of indicators selection**

| **Dimension** | | | **Variable** | | **Reference** |  |
| --- | --- | --- | --- | --- | --- | --- |
| OOP | X_1_: Average Out-of-pocket Payment (OOP) | | | | OOP will make families face catastrophic expenditure and become poor. A study in Nigeria confirmed that OOP increased the number of poor people by 0.8%[1]. |  |
| Socioeconomic factors | | X_2_: Gross Domestic Product (GDP) | | GDP has always been a primary measure of the economy[2]. Higher prices, use of expensive technology, and service availability are all associated with high CHE[3]. | | |
| Air pollution | | X_3_: Annual average PM_2.5_ concentration in the one year before the survey (PM_2.5_) | | PM_2.5_ is a primary indicator for potential air pollution caused by traffic and industry, inducing more medical expenses[4, 5]. | | |
| Health Service Demand | | X_4_: Proportion of Population Aged 65 or Over | | The per capita medical expense of people aged 65 or over is much higher than that of young people, although due to the gradual ageing of the population, this impact may not be enough to make ageing the main driver of health costs [6]. However, it should be considered since this study uses the open-access database CHARLS, which primarily consists of the middle-aged and elderly population. | | |
|  |  | X_5_: Prevalence of Non-communicable Diseases (NCDs) | | Households with at least one non-communicable disease are more likely to suffer from catastrophic health expenditure than the regional average[7]. | | |
|  |  | X_6_: Prevalence of Disability (Disability) | | Compared with non-disabled people, the health expenditure, out of payment and burden of disabled people are higher and have been significantly increased [8]. | | |
| Health Service Provision | | X_7_: Number of Nurses Per Thousand Persons | | Nurses are often the first or only medical workers when people seek medical and health services. They are at the forefront of public health promotion, prevention projects and intervention measures. When the number of patients cared for by each nurse decreased by one, the mortality within 30 days, the readmission rate within seven days after discharge decreased by 7%, and the length of hospitalization decreased by 3%[9], indicating less medical expense. | | |
| Health Policy | | X_8_: Health Insurance Coverage | | Despite the heterogeneity of economic protection capacity among different types of insurance, the universal coverage of medical insurance helps to reduce the incidence of catastrophic health expenditure in the region[10] | | |

**Appendix Table 2. Comparison of GTWR model without geographical subdivisions and that with it**

|  | **GTWR model without geographical subdivisions** | **GTWR with geographical subdivisions** |
| --- | --- | --- |
| **Bandwidth** | 42 | 42 |
| **Residual Sum of Square** | 577.243 | 492.273 |
| **AICc** | 634.531 | 622.243 |
| **R^2^** | .729 | .769 |
| **Adjusted R^2^** | .708 | .749 |

Note: GTWR, geographical and temporal weighted regression; OLS, ordinary least squares; Two models compared are completely consistent except whether they contain geographical subdivisions. GTWR with geographical subdivisions is the GTWR model finally adopted in this study.


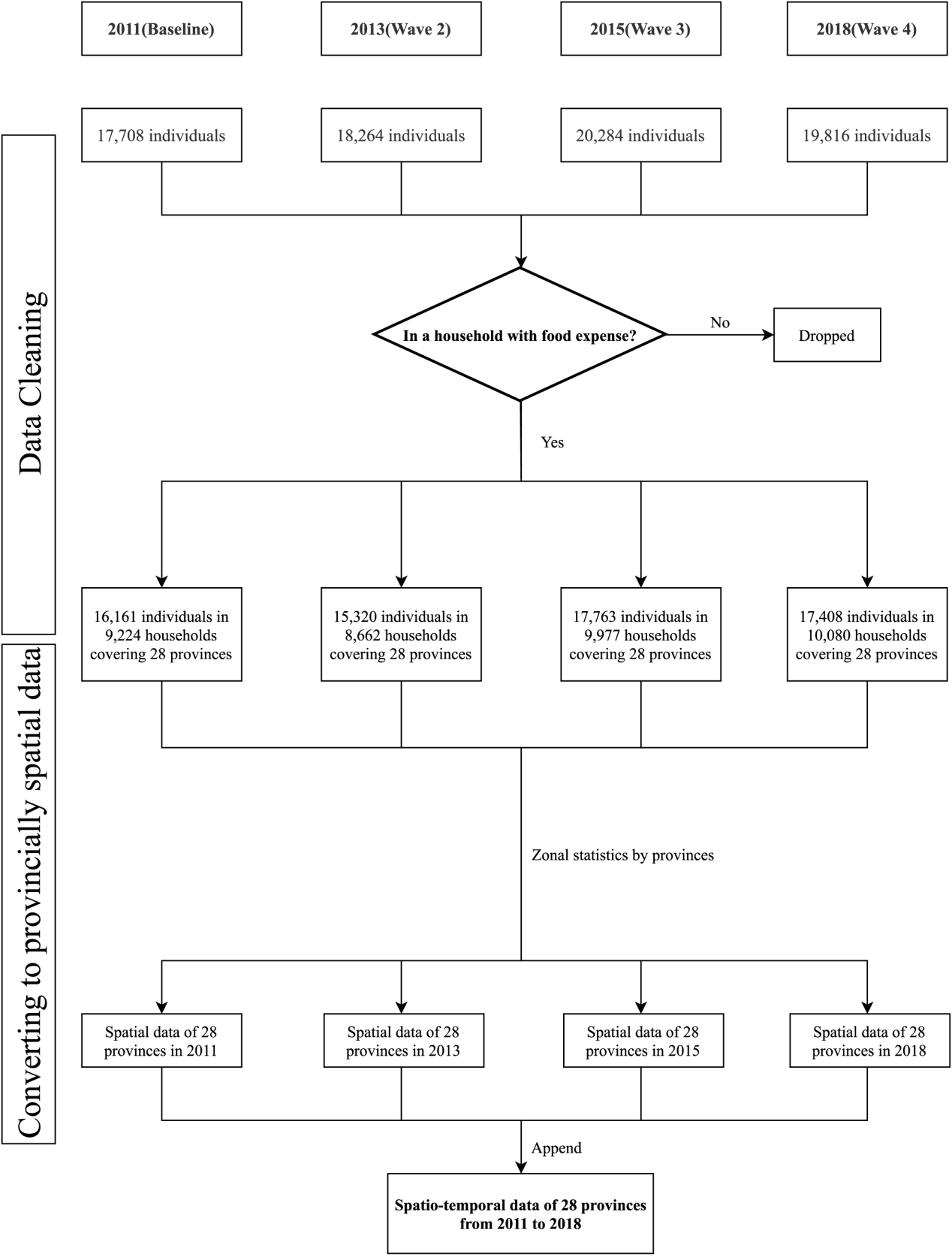


**Appendix Figure 1. Flowchart of data process in this study**

**Reference**

1. Aregbeshola BS, Khan SM. Out-of-pocket payments, catastrophic health expenditure and poverty among households in Nigeria 2010. International journal of health policy and management. 2018;7:798.

2. Coyle D. GDP. Princeton University Press; 2015.

3. Wagstaff A, Flores G, Hsu J, Smitz M-F, Chepynoga K, Buisman LR, et al. Progress on catastrophic health spending in 133 countries: a retrospective observational study. The Lancet Global Health. 2018;6:e169–79.

4. Johnston FH, Borchers-Arriagada N, Morgan GG, Jalaludin B, Palmer AJ, Williamson GJ, et al. Unprecedented health costs of smoke-related PM2. 5 from the 2019–20 Australian megafires. Nature Sustainability. 2021;4:42–7.

5. Xie Y, Dai H, Dong H, Hanaoka T, Masui T. Economic impacts from PM2. 5 pollution-related health effects in China: a provincial-level analysis. Environmental science & technology. 2016;50:4836–43.

6. Reinhardt UE. Does the aging of the population really drive the demand for health care? Health Affairs. 2003;22:27–39.

7. Kien VD, Van Minh H, Giang KB, Dao A, Ng N. Socioeconomic inequalities in catastrophic health expenditure and impoverishment associated with non-communicable diseases in urban Hanoi, Vietnam. International Journal for Equity in Health. 2016;15:1–11.

8. Mitra S, Findley PA, Sambamoorthi U. Health care expenditures of living with a disability: total expenditures, out-of-pocket expenses, and burden, 1996 to 2004. Archives of physical medicine and rehabilitation. 2009;90:1532–40.

9. Ullman AJ, Davidson PM. Patient safety: the value of the nurse. The Lancet. 2021;397:1861–3.

10. Li Y, Wu Q, Xu L, Legge D, Hao Y, Gao L, et al. Factors affecting catastrophic health expenditure and impoverishment from medical expenses in China: policy implications of universal health insurance. Bull World Health Org. 2012;90:664–71.
