# Supplementary figures and images for: Factors that dynamically affect provincial incidences of catastrophic health expenditure among middle-aged and elderly Chinese population-transition of disease financial risk protection from global to local
Source: BMC Geriatr. 2022 Sep 16;22:759. doi: 10.1186/s12877-022-03432-6 (PMC9479304; doi:10.1186/s12877-022-03432-6)

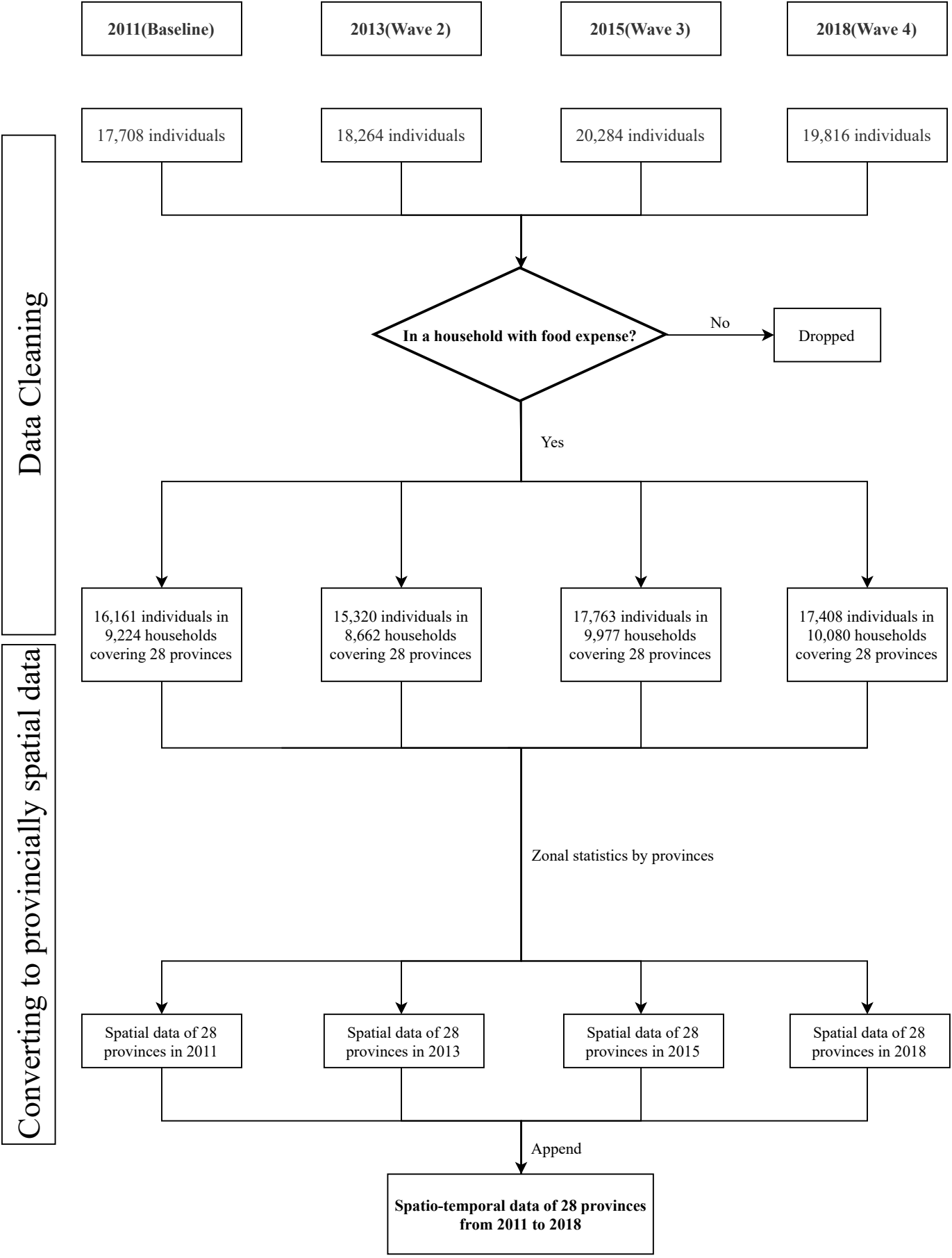

Supplement: Supplementary file 1 — Additional file 1: Appendix Table 1. Reference of indicators selection. Appendix Table 2. Comparison of GTWR model without geographical subdivisions and that with it. Appendix Figure 1. Flowchart of data process in this study. [file 12877_2022_3432_MOESM1_ESM.zip › Appendix Figure 1.pdf]
